# Comparison of the effects of itraconazole and prednisolone on *fibroblast growth factor*-2 gene expression and clinical manifestations in patients with persistent severe asthma

**DOI:** 10.22034/CMM.2023.345036.1401

**Published:** 2023-06

**Authors:** Mahsa Manafi Varkiani, Majid Mirsadraee, Zahra Anhaee Nasseri, Mohammadreza Khakzad, Shadi Ghaffari, Tayebeh Rabbani Nia

**Affiliations:** 1 Innovative Medical Research Center, Faculty of Medicine, Islamic Azad University, Mashhad Branch, Mashhad, Iran; 2 Department of Internal Medicine, Faculty of Medicine, Islamic Azad University, Mashhad Branch, Mashhad, Iran; 3 Department of Physiology, Faculty of Biology, Islamic Azad University, Damghan Branch, Damghan, Iran

**Keywords:** Asthma, Bronchial wall thickness, Fibroblast growth factor 2, Itraconazole, Prednisolone, Remodeling

## Abstract

**Background and Purpose:**

Considering the possible role of fungal sensitization in the treatment of resistant asthma, which may lead to the remodeling of bronchial structure, we theorized that itraconazole could result in better control of asthma. In this regard, this study aimed to compare the effects of itraconazole and prednisolone (routinely prescribed) on clinical, structural, and biomarker findings of the remodeling of asthma.

**Materials and Methods:**

This double-blind controlled randomized clinical trial was performed on 70 adult patients suffering from severe persistent asthma. The intervention group received 200 mg of itraconazole per day, and the control group received 10 mg of prednisolone per day, for 32 weeks, in addition to the classic treatment of asthma. The subjects were randomly divided into two groups, and assigned by sealed envelope. Blinding was performed by repacking the drug in a similar container. Primary outcomes were asthma control test score, fibroblast growth factor 2, and wall area percentage on RB1 bronchus measured by computed tomography. The outcomes were compared in subjects classified as allergic, eosinophilic, T2 low asthma, and four types of inflammatory cell classification in sputum.

**Results:**

Seventy subjects finished the 32-week trial (35 subjects in each group). Baseline data did not show significant differences between groups. A comparison of asthma variants showed significantly more severe cough and dyspnea in the allergic variant and higher spirometry results in T2-low asthma. Sputum cytology revealed a mixed pattern as the most frequent type (47%). After the trial, two groups improved in many parameters; however, FGF-2 improved more significantly by itraconazole (4.66±16.92 decreased to 1.14±2.98), and FEV1/FVC was significantly higher in the itraconazole group, compared to the control group. These results did not change in terms of asthma variants and sputum classification.

**Conclusion:**

Itraconazole was superior to prednisolone in the treatment of many clinical and spirometry aspects in severe persistent asthma.

## Introduction

Severe persistent asthma is an important subgroup of asthma that does not respond to two controller drugs, especially a combination of inhaled corticosteroids and long-acting beta 2 agonists. Unfortunately, these patients face a long-term decline in lung function, irreversible airway remodeling, and increased risk of life-threatening exacerbations [ 1 ]. It was shown that some of these patients did not respond to corticosteroids, anti-IL-5, and anti-IL-4/13 [ 2 ]. Sensitization to fungi, such as *Aspergillus* species, is considered an important risk factor for severe persistent asthma [ 3 ].

Sensitization to fungi is observed in about 70% of patients with severe asthma and is responsible for 15-48% of worse asthma control [ 4 ]. However, they rarely fulfill the diagnosis of allergic bronchopulmonary aspergillosis (ABPA), a florid response to airway infection with *Aspergillus* spp. In this condition, persistent endo-bronchial colonization with fungi, especially *Aspergillus fumigatus*, has proven to be associated with higher rates of radiological abnormalities, worse spirometry parameters, and lower reversibility to short-acting bronchodilators [ 5 ]. Therefore, reduction of the fungal burden of the airway using antifungal treatment may result in improved asthma control, lung function, and symptoms, which cannot be achieved by drugs, such as oral corticosteroids or other medicines recommended in the fifth step of the GINA strategy for the treatment of asthma [ 6 ]. Therefore, we believe that in case of the presence of an effective pathogen, such as fungi, anti-inflammatory agents are not successful and eradication of the offending is the treatment of choice. 

Airway remodeling is a well-known complication of chronic uncontrolled asthma. Classically, remodeling is characterized by histopathology; however, computed tomography (CT) and measurement of thickness or internal diameter of the right upper lobe main bronchus (RB1) are the best available non-invasive, biometric methods for the detection of airway remodeling. Our recent study about remodeling of the bronchial wall, evaluated by computed tomography, showed improvement in wall thickness percentage (wall thickness per outer radius×100) and inner lumen area by a course of treatment with itraconazole for 8 months [ 7 ]. 

These findings proved the involvement of fungal disease in the pathogenesis of asthma remodeling. However, differentiation between the cellular infiltration and structural change, including hyperplasia of the smooth muscle or mucosal gland, was not possible [ 5 ]. Therefore, the Fibroblast growth factor-2 (FGF-2, also known as basic fibroblast growth factor, b FGF) was added to our study, due to its dual action on inflammation and airway structural compartments. Previous studies have confirmed a significant correlation between FGF-2 levels and the severity of asthma. The FGF-2 links the airway inflammation and airway structural cells, like airway epithelial cells and smooth muscle. The FGF-2 has proved to contribute to smooth muscle hyperplasia [ 8 ]; therefore, it is considered a good marker of remodeling due to chronic inflammation following asthma [ 9 ]. It has been used as a marker of the activity of the remodeling [ 10 ] in sputum [ 11 ] and broncho-alveolar lavage [ 12 ].

We believe that if the etiology of bronchial inflammation is due to stimulation of epithelial cells by an offending agent, such as *Aspergillus* conidia or hypha, treatment will be more effective with the eradication of fungal infection, rather than suppression of inflammation by corticosteroid therapy, which is the current routine treatment of allergic bronchopulmonary Aspergillosis. The difference between these two treatments on airway remodeling can be evaluated by computed tomography and FGF-2 assessments.

This study aimed to evaluate the efficacy of eradication of fungal infection on airway remodeling due to severe persistent asthma, determined by imaging and FGF-2 expression in the sputum, as the best available marker of airway remodeling. The ancillary target of this study was to determine the accuracy of FGF-2 for the diagnosis of airway remodeling, considering computed tomography as the gold standard.

This study did not focus on any special fungal lung disease, such as severe asthma with fungal sensitization or Fungal bronchitis (heavy colonization of bronchi without elevation of specific IgE).

## Materials and Methods

### Pretrial phase

All new asthmatic subjects aged more than 18 years old, who were referred to a lung subspecialty clinic in Mashhad, Northeast of Iran, from October 2019 to 20 August 2020, and did not require urgent interventions or hospital admission, were considered the study population. The diagnosis of asthma was confirmed based on clinical symptoms, the results of physical examination (wheezing on auscultation), and the results of the spirometry test (forced expiratory volume [FEV_1_] <60% or the ratio of FEV_1_ to forced vital capacity [FVC] less than 75%). As the first-line therapy, they were treated by inhaled corticosteroid (ICS) plus long-acting beta 2 agonist. In case of resistance to first-line therapy, the ICS was increased to the maximum dose, and at the third visit, a second controller drug, especially Montelukast or Tiotropium, was added. 

### Participants

Upon the pretrial phase, non-responder subjects were evaluated for other underlying disorders. In case of a lack of underlying diseases, they entered into phase 3 of the clinical trial. Airway remodeling was non-invasively confirmed by morphometric measurement of the diameter of the first generation of the right upper lobe bronchus [ 13 ].

All the patients were tested for fungal sensitization by skin prick test for A. fumigatus, A. niger, A. flavus, A. nidulans, and A. amstelodami performed by AllergoTek Kit (AllergoTek, Kuwait) and/ or ELISA test for positive results of anti-Aspergillus antibodies against A. fumigatus provided by FungiXpert® Fungus (1–3)-beta-d-Glucan Detection Kit (ERA Biology, Tianjin, China), total IgE (ELISA test performed by Pishtaz Kit, Iran), and blood eosinophil count. 

Any patient with sensitivity or drug interaction with itraconazole, pregnancy, breastfeeding, gastro-esophageal reflux disease, or other pulmonary diseases, and other organ failure were excluded from the study. Moreover, patients with vocal cord dysfunction (evaluated by spirometry), allergic bronchopulmonary aspergillosis (screened by a total serum level of more than 1,000 IU/ml and imaging), and inability to give sputum sample (for RNA analysis) were excluded from this study. Finally, the eligible participants were categorized into two groups of 35 subjects each using the permuted block randomization method, generated by STATA software.

### Intervention

This is a phase three randomized clinical trial, active comparator study. The intervention group received 200 mg itraconazole (100 mg capsules twice daily, according to the past clinical trials [ 14 ]) while the control group received 10 mg prednisone (5 mg capsules twice daily, according to GINA guidelines) for 8 months, in addition to the previous treatment of asthma. Prednisolone was selected for the control group as it was the only available approved drug in our region for subjects who entered the step 5 GINA guideline for the treatment of asthma. Itraconazole and prednisolone powders were purchased from Tehran Daru Pharmaceutical Company, Iran and Alborz Daru Pharmaceutical Company, Iran, respectively. 

Afterward, the prednisolone tablets were put in capsules similar to itraconazole. Both itraconazole and prednisolone were repacked in containers that were completely similar in shape, which could not be recognizable by the staff and the patients. The packages were prescribed to the patients based on the table of random numbers, by a colleague in the pharmacy. The physician, the nurse, the patients, and the statistician were unaware of the group allocations; the codes were uncoded only at the end of the study after the statistical analysis was completed. The inclusion criteria were similar for both groups. Performing the fungal sensitization tests was not obligatory for patients, and the results of the fungal test were not considered an inclusion criterion; i.e., both patients with negative or positive results of the fungal test were included in the study.

The side effects of the drugs were checked weekly by telephone calls. Lab exams, including fasting blood sugar, liver enzymes, and creatinine, were checked every other month. In case of any adverse effects, the patient was excluded from the study and received high-dose prednisone. Primary outcomes were FGF-2 gene expression level in the sputum, RB1 bronchus wall thickness percentage, and asthma control test (ACT) score. Other parameters, including clinical findings, spirometry, computed tomography, serology, and sputum parameters were considered secondary outcomes. Therefore, all subjects underwent computed tomography, blood sampling, and induced sputum. Cough and dyspnea severity were graded by methods described in the previous study [ 15 ]. Informed consent was obtained for all these procedures.

### Classification of subjects

The serum levels of IgE, Aspergillosis IgG, eosinophil count, and white blood cell count were recorded by classifying the asthma endotype. The subjects with blood eosinophil of more than 300 ml and/or fractional exhaled nitric oxide (FeNO) of more than 20 ppm were considered TH2 high with subgroups of 1) allergic which showed IgE more than 150 mg/ml and eosinophil less than 1,000/ml and 2) eosinophilic that had FeNO more than 50 PPM and eosinophil more than 1,000/ml. A sub-classification was also performed for the eosinophilic group in which all subjects with positive results of IgG anti-aspergillosis, specific IgE, or skin prick test were classified as “allergic fungal airways disease (AFAD)”, while all subjects who were negative for the mentioned lab tests were considered “non-AFAD”. Subjects with low FeNO and eosinophil count (less than 150/mm3) were considered the low T2 group. Materials and Methods

A nebulizer using 2 ml of hypertonic saline (7%) was employed to induce and collect the sputum sample. The sputum samples were kept at -20 °C in the freezer in the clinic and sent to the research center as soon as possible to measure the expression level of the FGF-2 gene in the sputum (using real-time polymerase chain reaction) and cytology (eosinophil and neutrophil count). Sputum eosinophil of more than 3% was considered eosinophilic, while neutrophil of more than 76% was considered neutrophilic; moreover, none of them was considered paucigranulocytic and both of them were considered a mixed pattern. 

The NObreath® (Bedfont Scientific London, UK) was used to measure the exhaled nitric oxide (FeNO) levels during a 12-second breath. This device has an accuracy of 5ppb and measures 10 mL with 0.025 l/s flow. MicroMedical (Superspiro, London, UK) was used for the measurement of spirometry parameters, including FEV_1_, FVC, FEV_1_/FVC, forced expiratory flow (FEF)_25-75_, and FEF_25-75_/FVC. This device has an accuracy of 3% and a resolution of 10 mL volume 0.0251 l/s flow. 

Bronchial dimension was evaluated by a 16-slice CT scan (32-slice reconstruction) (Siemens company, Germany), and bronchial dimensions were measured in the RB1 bronchus in each patient, before and after the study. MarcoPacs software (version 3.1) was used for the bronchial dimension measurements, which were performed using a ×50 magnification ruler, by a method discussed before [ 16 ]. Airway wall thickness was measured according to bronchial wall thickness (internal and external), wall area percentage (ratio of whole wall area surface to whole bronchial surface), and the ratio of bronchial wall thickness to external bronchial diameter. The measurements were performed by two expert radiologists, and the mean values were used as the final number. To minimize the effect of the weight and height of patients on the measurements, all numbers were divided by body surface area.

Sputum purification was performed by 0.1% dithiothreitol to break the disulfide bonds of saliva. A small part of the suspension was used for a thin smear layer. Smears were stained with Hematoxylin-Eosin for cytology. The TRIzol named EX6101-RNX Plus Solution for total RNA isolation (Sinaclon CO, Iran) was used for RNA extraction. Then, cDNA was synthesized using a reverse transcription kit (Parstous Co, Iran). Furthermore, real-time quantitative-polymerase chain reaction (RT-qPCR) was performed using an SYBR Green-based qPCR kit (Parstous Co, Iran) and amplified on a CFX RT-PCR detection system (Corbett, USA). The primer sequences used in this study were designed through Primer Blast in the NCBI site and then produced by Sinaclon Co. The 2 ^-ΔΔCt^ method (Fold change) was used to calculate the expression of genes. 

Primer sequences included GAPDH-human (F): ATGGGGAAGGTGAAGGTCG, GAPDH-human (R): G GGGTCATTGATGGCAACAATA, Fibroblast Growth Factor 2, (F): AGAGCGACCCTCACATCAAG, Fibroblast Growth Factor 2, and (R): CCAGTTCGTTTCAGTGCCAC. A treatment card was provided for all patients, on which the future visits were recorded. Moreover, the cell phone number of the researcher was provided on the card for emergency situations.

### Statistical analysis

The sample size of the study was calculated at 70 with 35 subjects in each group, based on the calculated effect size of 0.7, the study power of 80%, and the significance level of 0.05. For reporting the results of descriptive analysis, the mean, ratio, and standard deviation values were used. The distribution of variables was evaluated using the Shapiro-Wilk test, skewness and elongation tests, and q-q plots. To evaluate the effect of the interventions, Mc Nemar and paired t-tests were used. As the comparison groups were not homogeneous in terms of confounding variables, a regression model was used to eliminate confounders; moreover, the beta coefficient and odds ratio (OR) were reported. *P* values of less than 0.05 was considered significant, for all tests. Moreover, the statistical analyses were performed using STATA software (version 14.1).

### Ethics approval and consent to participate

The study was approved by the Ethics Committee of the Central Organization of Islamic Azad University of Mashhad (IR.IAU.MSHD.REC.1399.088) and registered in the Iranian Registry of Clinical Trials  under the registration number IRCT20181225042116N1.

## Results

**Figure 1:**
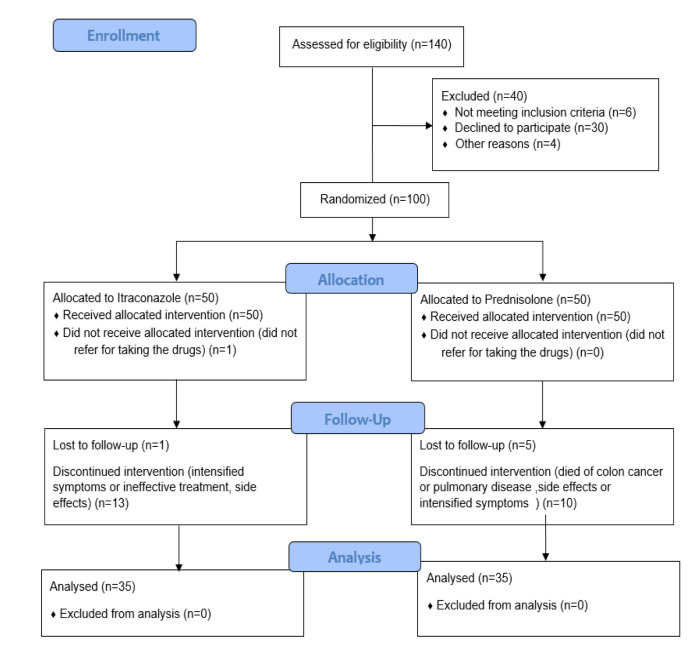
Flow diagram for screening and enrollment of patients for the present study

In total, 97 patients were enrolled in this study after the pretrial selection process, and 70 patients could complete 32 weeks of treatment with itraconazole and prednisolone (35 in each group) (Figure 1). Female gender was more frequent, and smoking, obesity, and anemia were reported in a proportion of subjects (Table 1). However, a comparison of the baseline data showed no significant difference between the two study groups in terms of mean age, gender distribution, smoking status, mean body mass index (BMI), duration of asthma, FeNO, and mean values of blood IgE, IgG, hemoglobin, eosinophil, and total white blood cell count (Table 1). Smoking status, hemoglobin, and BMI were taken into account as confounding variables. 

**Table 1:** Baseline characteristics of the severe persistent asthma subjects, including comparison of the two intervention groups.

Variable	Categories	Abnormal Count (percent) Range	Total	Itraconazole (N=35)	Prednisolone (N=35)	*P*-value
Age (years)		-	56.67±12.58	55.74±13.70	57.60±11.48	0.42^‡^
Gender N (%)	Male	-	29 (41%)	14 (40)	15 (42.56)	0.78†
	Female	-	41 (59%)	21 (60)	20 (57.14)	
Smoking N (%)	Never	-	48 (68%)	24 (68)	24 (68)	0.54†
	Ex-smoker	-	9 (12%)	4 (11)	5 (14)	
	Current smoker	-	13 (20%)	7 (20)	6 (17)	
BMI (kg/m^2^)		47/70 (68%) 14-38	27.49±6.04	28.22±5.85	26.75±6.22	0.37^‡^
Duration of asthma (months)		2-40	12.07±9.31	11.68±9.47	12.45±92.6	0.915^‡^
Serum IgE (IU/mL)		47/64 (74%) 27-900	228±48.01	281±42.61	176±53.42	0.09^‡^
IgG *Aspergillus* (mg/dL)		37/62 (60%) 3-102	28.62±11.42	34.67±10.36	20.78±12.48	0.06^‡^
FeNO (PPM)		70/70 (100%) 20-142	45.84±2.40	45.77±26.69	45.91±21.53	0.98
Eosinophil count (cells/mcL)		8/64 (12%) 20-2500	4.71±1.79	5.64±1.23	3.87±2.36	0.07^‡^
White blood cell count (cells/L)		14/64 (19%) 6-15	8.93±2.87	9.34±2.28	8.53±3.47	0.09^‡^
Hemoglobin (G/dl)		14/64 (22%) 9-1747/64	13.5±1.91	13.44±1.70	13.6±2.11	0.47
Sputum eosinophil (>3%)		49/70 (70%) 2-14	6.75±3.92	6.87±4.20	6.64±3.67	0.80
Sputum neutrophil (>67%)		50/70 (71%) 62-86	71.77±6.50	70.68±6.77	72.86±6.13	0.16

The results of regression analysis,

† The results of the Chi-square test, BMI: body mass index

Overall, the clinical findings of subjects showed severe symptoms, low ACT scores, frequent asthma attacks, and hospital admission (Table 2). Classification of variants of asthma showed that allergic-type was the most prevalent (n=41, 65%), followed by eosinophilic (n=13, 20%) and low T2 (n=10, 15%) (Table 3). FeNO was more than 20 PPM in all of the subjects; therefore, FeNO values of 20-35, 35-50, and above 50 PPM were considered T2 low, allergic, and eosinophilic, respectively. A comparison of these variants showed significantly more severe cough and dyspnea in the allergic variant, and higher spirometry results in T2 low asthma; however, other parameters did not show significant differences (Table 3). Among T2 high subjects, half of them (n=28, 49%) revealed the criteria of AFAD with an average of 46±22 mg/ml for anti-Aspergillosis IgG. Sputum cytology classification showed a mixed pattern as the most frequent form (n=34, 48%), followed by neutrophilic (n=19, 27%), eosinophilic (n=14, 20%), and paucigranulocytic (n=3, 5%) (Table 4). 

**Table 2:** Comparison of clinical findings of itraconazole and prednisolone groups, after 32 weeks of treatment, in severe persistent asthma.

Findings	Score	Total	Before		After	
			Itraconazole	Prednisolone	Itraconazole	Prednisolone
Cough severity	No	0 (0%)	0 (0%)	0 (0%)	4 (11.43%)*
	4 (11.43%)‡Mild, not daily	0 (0%)	0 (0%)	0 (0%)	11 (31.43%)*	10 (28.57%)‡
	Moderate, daily	12 (17.14%)	6 (17.14%)	6 (17.14%)	11 (31.43%)*
	11 (31.43%)‡Severe	39 (55.71%)	20 (57.14%)	19 (24.29%)	1 (2.86%)*	2 (5.71%)‡
	Continuous	19 (27.14%)	9 (25.71%)	10 (28.57%)	0 (0%)*	1 (8%)‡
Dyspnea Severity	No	0 (0%)	0 (0%)	0 (0%)	0 (0%)	0 (0%)
	Mild, not daily	0 (0%)	0 (0%)	0 (0%)	14 (40%)*	12 (34.29%)‡
	Moderate, daily	3 (4.29%)	2 (5.71%)	1 (2.86%)	9 (25.71%)*	11 (31.43%)‡
	Severe	37 (52.86%)	21 (60%)	16 (45.71%)	7 (20%)*	6 (17.14%)
	Continuous	30 (42.86%)	12 (34.29%)	18 (51.43%)	5 (14.29%)*	6 (17.14%)‡
Sputum	-	70 (100%)	35 (100%)	35 (100%)	29 (82%)	29 (82%)
Sputum type	None	0 (0%)	0 (0%)	0 (0%)	6 (17.14%)*	6 (17.14%)‡
	Transparent	0 (0%)	0 (0%)	0 (0%)	10 (28.57%)*	7 (20%)‡
	White	32 (45.71%)	19 (54.29%)	13 (37.14%)	12 (34.29%)*	14 (40%)‡
	Green	38 (54.29%)	16 (45.71%)	22 (62.86%)	7 (20%)*	8 (22.86%)‡
	Purulent	0 (0%)	0 (0%)	0 (0%)	0 (0%)	0(0%)
Nasal grip	-	35 (50%)	18(51.34%)	17(48.57%)	12(34.29%)	9(25.71%)‡
ACT score		12.12±3.24	12.25±3.10	12 ±3.42	17.65±5.05*	17.05±5.19‡
Hospital admission	During 32 weeks	1.08±1.43%	1.14±1.53%	1.02±1.33%	0.45±0.88%*	0.74±1.09%
Asthma attack	During 32 weeks	5.64±2.75	5.4±2.62	5.88±2.89	2.91±2.00* ƚ	4.34±2.95‡
wheezing	During 32 weeks	70(100%)	35(100%)	35(100%)	9(25.71%) * ƚ	18(51.43%) ‡

* = significant difference in the itraconazole group before and after the trial

ƚ = Significant difference between itraconazole and prednisolone groups

‡ = Significance difference in prednisolone group before and after the trial

ACT: asthma control test

**Table 3:** Comparison of para-clinical findings between itraconazole and prednisolone groups.

Lab Findings	Unit	Before		After	
		Itraconazole	Prednisolone	Itraconazole	Prednisolone
FeNO	PPM	45.77±26.69	45.91±21.53	33.8±31.28*	33±25‡
Sputum Eosinophil	%	6.87±4.20	6.64±3.67	5.27±3.77*	4.66±3.05‡
Sputum Neutrophil	%	70.68±6.77	72.86±6.13	72.86±6.93	73.88±7.07
FGF-2	Fold change	4.66±16.92	7±23.41	1.14±2.98* ƚ	3.49±7.70
FVC %pred.	%	59.91±19.04	61.08±18.05	71.91±19.65*	70.28±19.94‡
FEV1%pred.	%	50.54 ±18.16	52.14±15.03	66.62±23.62*	64.6±22.91‡
FEV1/FVC	%	70.34 ±15.13	70.95 ±12.01	84.01 ±37.15* ƚ	71.41±11.84‡
FEF25-75%pred	%	39.17 ±20.84	38.65 ±19.53	61.22 ±34.77*	58.40 ±32.67‡
Outer diameter	Mm	4.14±0.20	4.12±0.17	4.07±0.17*	4.07±0.13 ‡
Inner diameter	Mm	2.27±0.19	2.27±0.19	2.36±0.19*	2.37±0.23‡
Wall thickness	Mm	0.93±0.13	0.92±0.11	0.85±0.14*	0.84±0.11‡
Wall area%	%	69.58±5.55	69.22±4.75	65.87±7.18*	65.71±6.04‡
Wall thickness/outer diameter	%	0.22±0.02	0.22±0.01	0.20±0.03*	0.20±0.02‡

* = significance difference between before and after the trial in itraconazole group

ƚ = Significant difference between itraconazole and prednisolone groups

‡ = Significance differences between before and after the trial in prednisolone group

FVC; forced vital capacity, FEV1; the first second of forced expiration, FEF; forced expiratory flow, FEF_25-75_; forced mid-expiratory flow, FeNO: Expiratory fraction of nitric oxide, FGF-2= Fibroblast growth factor-2

**Table 4:** Comparison of selected asthma parameters between sputum cytology patterns in subjects.

Parameters	Groups	Before			After		
		Allergic	Eosinophilic	Low T2	Allergic	Eosinophilic	Low T2
ACT (Score)	ITC	10.9±2.7	13.4±3.7	13.0±2.2	17.5±5.2 ^*^	18.8±4.4 ^*^	17.4±7.5
	Pred	11.4±3.4	12.0±4.3	13.0±2.2	17.2±5.5 ^‡^	17.0±3.6 ^‡^	16.4±6.9
FeNO	ITC	40.2±10.8	76.2±56.4	28.00±7.3	24.7±12.7^*^	66.0±56.7^*^	23.6±19.2
	Pred	43.2±11.5	74.3±33.1	25.4±7.4	28.6±15.9^‡^	55.6±45.9^‡^^ƚ^	24.8±18.6
FGF-2	ITC	6.3±3.2	1.8±2.9	1.3±1.9	0.1±0.1 ^*^	2.3±4.5	0.2±0.2 ^*^
	Pred	10.0±2.4	2.0±1.7	1.4±2.0	0.5±0.4 ^‡^	0.4±0.4	0
FEV1%pred.	ITC	41.8±17.8	52.1±14.9	65.6±5.1	62.1±26.8 ^*^	73.1±17.1 ^*^^ƚ^	70.2±29.0
	Pred	50.0±156	46.3±14.1	61±7.5	63.5±25.9 ^‡^	64.0±15.3 ^‡^	67.0±24.5
FEV1/ FVC	ITC	69.2±17.1	75.1 ±18.4	68.2 ±4.9	72.3±12.2	77.5±15.5 ^ƚ^	69.0±3.3
	Pred	73.7±12.4	60.6±11.0 ^ƚ^	71.8±7.7	75.7±9.5	65.8±13.5	78.0±15.1^ƚ^
Inner diameter	ITC	2.2±0.1	2.3±0.1	2.3±0.2	2.3±0.1	2.5±0.1	2.3±0.2
	Pred	2.2±0.1	2.3±0.2	2.3±0.1	2.3±0.2	2.5±0.1	2.3±0.2
Wall area%	ITC	71.2±5.3	68.0±5.8	64.8±4.9	67.2±6.9	59.8±6.5 ^*^	64.6±7.5
	Pred	69.4±4.1	70.3±7.1	66.3±4.0	66.4±6.3	61.9±6.7 ^‡^	64.4±6.5

ITC: Itraconazole, Pred: Prednisolone, ACT: asthma control test, FVC; forced vital capacity, FEV_1_; the first second of forced expiration, FeNO: Expiratory fraction of nitric oxide, FGF-2= Fibroblast growth factor2

### Results of the intervention

After 32 weeks of treatment, clinical findings, including cough and dyspnea severity, sputum type (Supplementary file 1), and wheezing improved significantly in both groups; however, itraconazole disappeared wheezing significantly more than prednisolone (Table 5). Asthma attack and hospital admission decreased significantly in both groups, although, asthma attack was significantly lower in the itraconazole group than prednisolone group (Table 5). The nasal grip was not affected by itraconazole, but prednisolone showed significant improvement. A comparison of the itraconazole and prednisolone group, in terms of sputum cytology classification, did not show a significant difference between classes of cytology (Table 3, Supplementary file 2).

**Table 5:** Comparison of selected asthma parameters between sputum cytology patterns of the subjects.

Parameters	Groups	Before				After			
		Eosinophilic	Neutrophilic	PGA	Mixed	Eosinophilic	Neutrophilic	PGA	Mixed
ACT (Score)	ITC	12.2±3.8	11.3±3.2	14.6±1.1	12.3±2.5	20.2±3.9 ^*^^ƚ^	16.2±5.7 ^*^^ƚ^	17.3±0.5	16.9±5.6 ^*^
	Pred	12.5±2.8	9.6±3.6	NA	13.0±2.8	16.5±5.0 ^‡^	14.6±4.1^‡^	NA	18.3±5.1 ^*^
FeNO	ITC	43.5±11.6	49.2±36.5	24.6±8.0	50.0±29.5	24.0±10.3 ^ƚ^	40.4±43.2	19.3±6.1	39.8±35.1
	Pred	38.7±14.9	55.8±33.9	NA	42.5±12.8	30.5±20.8	39.6±41.3^‡^	NA	30.3±14.1^‡^^ƚ^
FGF-2	ITC	3.6±4.2	2.0±2.4	0.9±0.7	8.0±27.7	1.1±2.9^*^	0.3±0.4^*^	3.4±5.2	1.2±3.4 ^ƚ^
	Pred	0.3±0.4	1.8±2.4	NA	10.7±29.8	0.5±0.4	0.4±0.4^‡^	NA	5.4±9.5^‡^
FEV1%pred.	ITC	52.9±17.6	50.1±17.6	62.0±9.1	46.9±17.1	76.8±18.8^*^^ƚ^	60.4±26.8^*^^ƚ^	69.0±7.0	62.5±25.7^*^
	Pred	40.5±25.9	47.1±14.1	NA	56.7±14.6	71.0±32.2 ^‡^	55.2±15.2	NA	67.8±23.8^‡^^ƚ^
FEV1/ FVC	ITC	73.4 ±18.0	65.3 ±12.1	72.3±7.1	71.1±16.4	77.0±6.7^*^	70.6±10.9	78.0±14.7	70.1±13.2
	Pred	76.8±14.9	66.6±12.3	NA	71.6±11.3	85.0±14.0^*^^ƚ^	74.3±16.1^*^	NA	72.3±8.4
Inner diameter	ITC	2.2±0.2	2.3±0.2	2.1±0.1	2.3±0.1	2.3±0.2	2.3±0.2	2.2±0.3	2.3±0.2
	Pred	2.1±0.1	2.2±0.1	NA	2.3±0.1	2.3±0.2	2.3±0.2	NA	2.4±0.2
Wall area%	ITC	70.2±4.9	68.6±7.2	70.8±2.6	69.3±5.6	64.8±6.8 ^*^	66.2±8.5	67.7±5.4	65.6±7.4
	Pred	73.5±4.0	71.0±5.0	NA	67.5±3.9^*^	67.1±6.1^‡^	67.0±5.7	NA	64.7±6.8

ITC: Itraconazole, Pred: Prednisolone, PGA: Paucigranulocytic, FVC; forced vital capacity, FEV1; the first second of forced expiration, FeNO: Expiratory fraction of nitric oxide, FGF-2= Fibroblast growth factor2

Preclinical investigations, including FeNO, inflammatory cells in sputum, Spirometry, and biometric measurements of RB1 were comparable in both groups before the trial (Tables 1 and 5). The basal FeNO and the sputum eosinophil were higher than normal (Tables 1 and 5), and spirometry showed a severe obstructive pattern (Table 5). After the trial, FeNO and sputum eosinophil were decreased significantly in both groups; however, FGF-2 decreased significantly by itraconazole, and it was significantly lower than that in the prednisolone group (Table 5). Spirometry and biometric measurements of RB1 bronchus showed significant improvement in both groups; however, FEV1/FVC was significantly higher in the itraconazole group, compared to the prednisolone group (Table 5). 

After the trial, allergic and eosinophilic variants showed significant improvement in ACT score, FEV1 percent predicted, and FeNO measurement (Table 3). The allergic variant showed significant improvement in FGF-2; in addition, the eosinophilic variant revealed a significant decrease in FGF-2 and FeNO in the prednisolone group (Table 3, Supplementary file 3). Considering sputum cytology classifications, comparison between itraconazole and prednisolone groups showed significant differences in terms of clinical findings, spirometry, FGF-2, and biometric measurements of RB1 (Table 4). Paucigranulocytic asthma was not detected in the prednisolone group after the trial. In the mixed pattern group, FGF-2 was significantly lower in the itraconazole group, compared to the prednisolone group (Table 4).

### Course of the study

In total, 14 patients in itraconazole and 15 patients in the prednisolone groups missed the follow-up during 32 weeks. Recorded side effects in the itraconazole group were gastrointestinal discomfort (vomiting, dyspepsia, and diarrhea) (n=5, 38%), skin rashes (n=3, 23%), and upper respiratory infection (n=2, 15%). Moreover, no response to treatment was observed in three subjects (23%). The side effects in the prednisolone group were glucose impairments (n=7, 70%), weight gain (n=2, 20%), pulmonary infection with other pathogens (n=7, 70%), and colon cancer (n=1, 10%).

## Discussion

The present study showed that the 32-week administration of 200 mg/day of itraconazole inhibited the gene expression of the FGF-2, increased the percentage of FEV1/FVC, and decreased asthma attacks and wheezing. The overall results of para-clinical findings, lung examination (wheezing on chest auscultation), and frequency of asthma attacks (evaluated based on statements of patients) suggest itraconazole is superior to prednisolone in patients with severe asthma, irrespective of the presence of fungal sensitization or infection. Among these parameters, we would like to highlight the increase in the FGF-2, ACT, and inner diameter of RB1 bronchus. However, itraconazole improved FGF-2 as an inflammatory marker significantly more than prednisolone, which indicates a more profound suppression of inflammation by the elimination of an etiological factor. This etiology should be the growth of fungi inside the epithelial cells and the release of its antigen, which leads to aggravation of asthma. Furthermore, this phenomenon could be suppressed by itraconazole and improve asthma.

 In our previous RCT study about the benefit of antifungal therapy on severe persistent asthma [ 5 ], the results showed a better score and the global effectiveness of treatment by administration of 400 mg/day itraconazole for 4 months, compared to the administration of 10 mg/day prednisolone. Furthermore, itraconazole suppressed most of the clinical symptoms in 60% of patients, while prednisolone was only able to control dyspnea. Therefore, both our previous and present studies indicate the efficacy of itraconazole in the treatment of asthma before beginning step 5 of the treatment of asthma

There are only a few studies on the efficacy of antifungal agents in patients with severe asthma without ABPA. In a study, Lin et al. [ 17 ] performed a 6-year follow-up on 73 patients with severe asthma, who consumed 200 mg/day of itraconazole for 3 months. The aforementioned study confirmed the efficacy of low-dose itraconazole on patients with severe asthma (improved lung function and acute exacerbations), although it was limited considering the lack of a control group for comparison. Furthermore, the observed effects were not the pure effects of itraconazole, considering the use of add-on therapies, including systemic steroids, anti-IgE therapy (Omalizumab), and long-acting muscarinic antagonists in patients.

In another study, Denning et al. [ 18 ] compared the efficacy of the administration of 400 mg/day of itraconazole and placebo for 8 months. They performed a 16-week follow-up on patients with severe asthma with fungal sensitization (SAFS) in terms of the Asthma Quality of Life Questionnaire (AQLQ) score, rhinitis score, total IgE, and respiratory function. The results of their study showed significant improvement in AQLQ and rhinitis scores, decreased levels of serum IgE, and improved morning peak expiratory flow (PEF) in the intervention group, without severe adverse events. 

Case reports have also confirmed the efficacy of itraconazole in patients with SAFS [ 19 ]. Follow-up and course of treatment showed that itraconazole had fewer adverse effects, and some subjects experienced a relapse of wheezing after discontinuation of itraconazole for a while. We believe that the resolution of disease in these patients is attributed to the reduced fungal load which have colonized the body of the patients, including the airways, sinuses, gut, and/or skin; However, colonization cannot be detected easily, as many airborne fungi established in the human host cannot be grown in laboratory conditions [ 20 ]. 

Another possible mechanism is the interaction between itraconazole and inhaled corticosteroids, such as budesonide and fluticasone, which lead to increased plasma concentration and half-life of inhaled corticosteroids (1.6 increased plasma concentration and 3.7-fold of terminal half-life for budesonide in one study [ 21 , 22 ]. However, the immunomodulatory effect of itraconazole (suppression of cytokines secretion, such as interleukins-4 and -5 and T cells) was controversial, and some investigators have rejected such an effect [ 23 ]. 

Given the different responses of patients with different diseases, further molecular studies are required to determine the mechanism of action for the efficacy of antifungal agents in patients with severe asthma. The FGF-2 was introduced as a remodeling biomarker of asthma severity, which is inversely correlated with the FEV_1_/FVC ratio [ 7 ]; therefore, it indicates that increasing the amount of FGF-2 accompanies more severe airway obstruction. The FGF-2 revealed mitogenic activity on structural cells of airways, such as epithelial cells, endothelial cells, and smooth muscles [ 9 ]. It was also proven to regulate angiogenesis [ 24 ] through the proliferation of endothelial cells and the release of various chemo-attracting mediators from endothelial cells, which are required for airway remodeling [ 25 ]. These two structural changes point toward the importance of FGF-2 for remodeling and its consequences which can be shown as bronchial wall thickening by imaging studies.

However, in another study, FGF-2 has been reported to be associated with significantly higher FEV1, which indicates lower non-fixed airway obstructive asthma; therefore, the authors consider FGF-2 a protective mediator, which may be considered a target for the treatment of asthma and chronic obstructive pulmonary disease [ 26 ]. Some drugs which target FGF-2 production, FGF-2 molecule (by auto-antibodies), and its receptors have been invented [ 9 ]. However, the results of the present study showed that after the treatment with itraconazole, the expression of the FGF-2 gene was suppressed. 

We believe that anti-fungal agents show a greater effect on the suppression of inflammatory mediators due to targeting the origin of the disease process. Nevertheless, FGF-2 has a converse effect on asthma; accordingly, some studies have indicated that FGF-2 promotes airway inflammation through the FGFR/MAPK/NF-κB pathway in the airway epithelial cells. Moreover, some reports have confirmed its immune regulation by establishing the integrity of structural cells [ 27 ]. Therefore, time is needed to determine how we can use the FGF-2 probing treatment for asthma. Tan et al. showed that FGF-2 is not dependent on IgE and eosinophilic inflammation of asthma [ 28 ], and also introduced neutrophilic inflammation via Th1 or Th17 as the potential mechanism of FGF-2. The subjects of the present study, who were resistant to inhaled corticosteroids, would fall in this category. The result of the present study revealed high levels of eosinophil and neutrophil in the sputum, 49% of which was classified as a mixed pattern.

One of the limitations of the present study was the small number of patients in the two study groups, which made subgroup analysis of low statistical value. In this regard, subjects with fixed airway obstruction were considered in this study; the subjects who could not be treated with drugs and needed bronchial thermoplasty. For the time being, no non-invasive method is available to differentiate non-responders from other subjects. Enrollment of patients from one clinic is another limitation of this study as it increases the chance of bias in the results; therefore, caution should be exercised for the generalizability of the results. 

The potential confounding factors in this study were that some subjects continued to smoke, and some of them suffered from obesity and anemia, but randomization was able to balance these factors between the two groups.

## Conclusion

The superiority of itraconazole on FGF-2, FEV1/FVC, asthma attacks, and wheezing on chest auscultation during the treatment period compared with prednisolone in patients with severe asthma step 5 demonstrated that itraconazole was better than prednisolone. Future studies with longer follow-up periods along with a greater focus on safety are required for definite conclusions about the appropriateness of using itraconazole for the treatment of patients with severe asthma. New diagnostic tools for the detection of the presence of fungal infection inside the epithelial cells may help select appropriate subjects for anti-fungal therapy.
